# The oral microbiome in autoimmune diseases: friend or foe?

**DOI:** 10.1186/s12967-023-03995-x

**Published:** 2023-03-22

**Authors:** Xiaoyan Huang, Xiangyu Huang, Yi Huang, Jiarong Zheng, Ye Lu, Zizhao Mai, Xinyuan Zhao, Li Cui, Shaohong Huang

**Affiliations:** 1 Department of Preventive Dentistry, Stomatological Hospital, School of Stomatology, Southern Medical University, Haizhu District, No.366 Jiangnan Da Dao Nan, Guangzhou, 510280 China; 2 Department of Endodontics, Stomatological Hospital, School of Stomatology, Southern Medical University, Haizhu District, No.366 Jiangnan Da Dao Nan, Guangzhou, 510280 China; 3 Department of Dentistry, The First Affiliated Hospital, Sun Yat-Sen University, Zhongshan 2nd Road, Guangzhou, 510080 China; 4 Department of Oral and Maxillofacial Surgery, Stomatological Hospital, School of Stomatology, Southern Medical University, Haizhu District, Guangzhou, 510280 China; 5 Division of Oral Biology and Medicine, School of Dentistry, University of California, Los Angeles, CA 90095 USA

**Keywords:** Oral microbiota, Homeostasis, Dysbiosis, Autoimmune diseases, Targeted therapies

## Abstract

The human body is colonized by abundant and diverse microorganisms, collectively known as the microbiome. The oral cavity has more than 700 species of bacteria and consists of unique microbiome niches on mucosal surfaces, on tooth hard tissue, and in saliva. The homeostatic balance between the oral microbiota and the immune system plays an indispensable role in maintaining the well-being and health status of the human host. Growing evidence has demonstrated that oral microbiota dysbiosis is actively involved in regulating the initiation and progression of an array of autoimmune diseases.

Oral microbiota dysbiosis is driven by multiple factors, such as host genetic factors, dietary habits, stress, smoking, administration of antibiotics, tissue injury and infection. The dysregulation in the oral microbiome plays a crucial role in triggering and promoting autoimmune diseases via several mechanisms, including microbial translocation, molecular mimicry, autoantigen overproduction, and amplification of autoimmune responses by cytokines. Good oral hygiene behaviors, low carbohydrate diets, healthy lifestyles, usage of prebiotics, probiotics or synbiotics, oral microbiota transplantation and nanomedicine-based therapeutics are promising avenues for maintaining a balanced oral microbiome and treating oral microbiota-mediated autoimmune diseases. Thus, a comprehensive understanding of the relationship between oral microbiota dysbiosis and autoimmune diseases is critical for providing novel insights into the development of oral microbiota-based therapeutic approaches for combating these refractory diseases.

## Background

The human microbiota, which consists of approximately 10–100 trillion symbiotic microorganisms, defines the sum of all microbes forming communities present in any habitat, including the skin, oral cavity, respiratory system, gastrointestinal tract, and urogenital tract [1]. Normally, the relationship between oral microbes and the host is dynamic and homeostatic. Due to the coevolution between the host and microbiota, the microbiota contributes to adjusting and maintaining human health [2]. On the other hand, components of the innate and adaptive immune subsystems interact and work together to eliminate harmful pathogens and selectively promote commensal tolerance [3]. However, any genetic or environmental change in the bilateral interaction between the microbiota and immune system can lead to pathological conditions such as infection, inflammation and autoimmune diseases [4, 5].

**Fig. 1:**
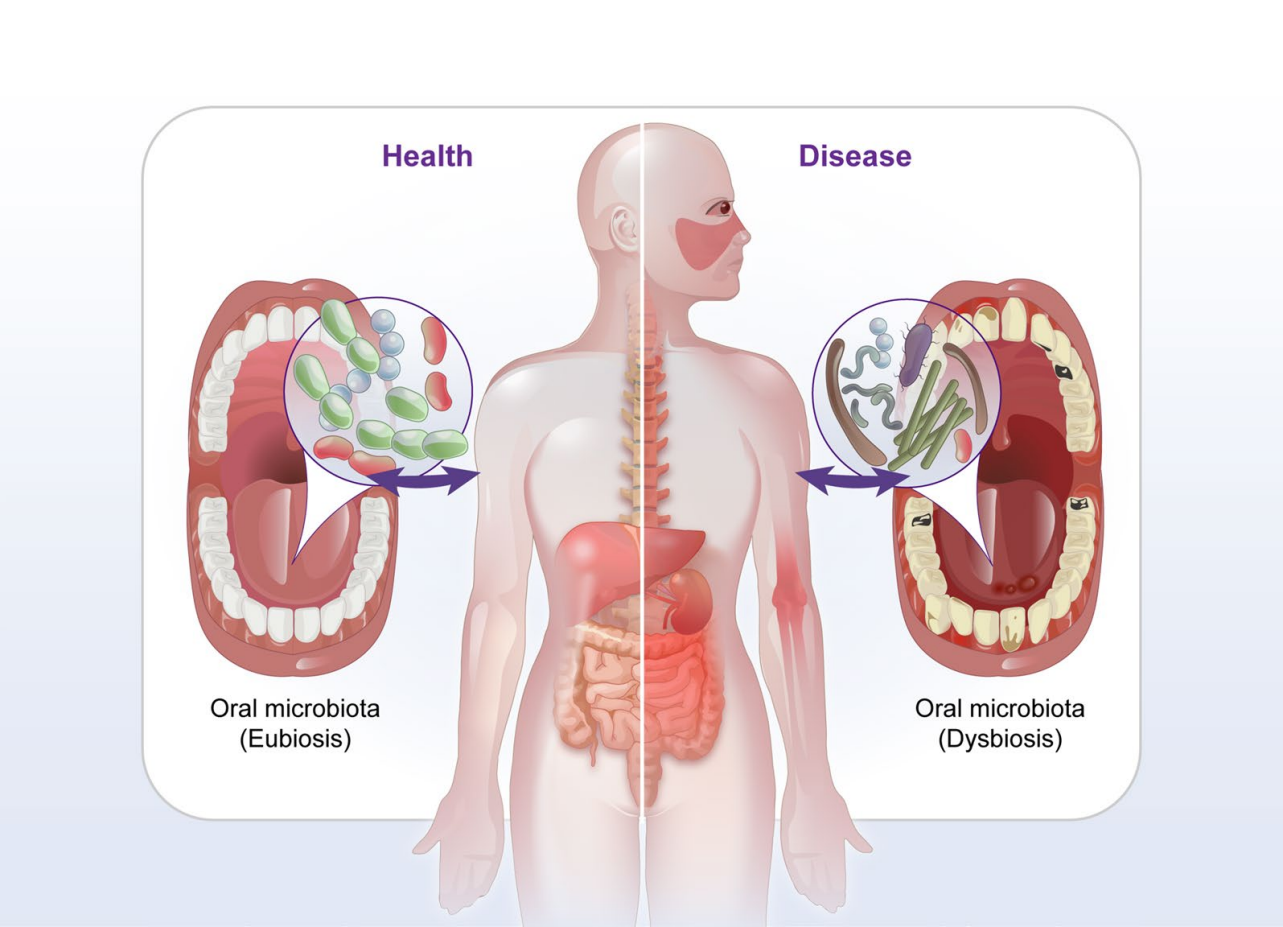
The bidirectional role of oral microbiota in the organism: eubiosis and dysbiosis. The eubiosis of oral microbiota contribute to homeostasis maintaining. Within microbiota dysbiosis, the homeostasis between the host and commensal is destroyed, accompany by abnormal immune response and leading to IBD, RA, SLE, SS, AS, MS, AILDs, IgAN. IBD, Inflammatory bowel disease; RA, Rheumatoid arthritis; SLE, Systemic lupus erythematosus; SS, Sjogren syndrome; AS, Ankylosing spondylitis; MS, Multiple sclerosis; AILDs, Autoimmune liver diseases; IgAN, Immunoglobulin A nephropathy

The oral cavity harbors the second largest and most diverse microbiota after the gastrointestinal tract, with 770 opportunistic, commensal, and pathogenic bacterial species [6]. Many unfavorable factors, such as smoking, administration of antibiotics and infections can significantly affect the composition and structure of the oral microbiota, resulting in oral diseases and systemic diseases [7]. Under the conditions of oral microbiota dysbiosis, certain commensal microorganisms in the oral cavity are transformed into pathogenic microorganisms, which are highly virulent and able to escape elimination by the immune system [8–10]. Accumulating evidence has revealed that oral microbiota dysbiosis might play a critical role in modulating the initiation and progression of a wide array of autoimmune diseases [11–14] (Fig. 1). For example, alterations in oral microbiota composition and structure were observed in mice with ligature-induced periodontitis, which may trigger increased secretion of the local gingival Th17 cells [15]. *Porphyromonas gingivalis* can transform arginine to citrulline in proteins via arginine gingipains and peptidylarginine deiminases (PADs) [16]. The immune system recognizes citrullinated proteins as autoimmune antigens and produces anti-citrullinated peptide antibodies, which subsequently activate the complement system and lead to Rheumatoid arthritis (RA) [17]. *Aggregatibacter actinomycetemcomitans* (*Aa*) is the key bacterium triggering autoimmune responses in RA, and it is a known periodontal pathogen that can secrete the pore-forming toxin leukotoxin-A (LtxA). LtxA induces the production of citrullinated antigen by abnormally activating citrullinating enzymes in neutrophils [18]. In addition, varying amounts of antibodies against different oral bacteria were detected in serum samples from patients with systemic lupus erythematosus (SLE). Higher antibody titers against *Aa, P. gingivalis, Treponema denticola,* and *Capnocytophaga. ochracea* were found in SLE patients positive for anti-dsDNA antibodies. In particular, antibodies against *Aa* were significantly associated with SLE disease activity [19].

Oral microbiome is readily accessible and easily sampled, thereby dissecting the composition and function of oral microorganisms is crucial for elucidating the underlying molecular mechanisms for the development of autoimmune diseases. The current review comprehensively covers the pathological changes affecting the bilateral cross-talks between oral microbiota and immune system, which might result in the initiation and progression of autoimmune diseases. We also discussed the major molecular mechanisms that oral microbiota dysbiosis affects autoimmune diseases (microbial translocation, molecular mimicry, autoantigen overproduction and amplification of autoimmunity by cytokines). The potential therapeutic strategies for balancing the oral microbiota are also focused on, which may provide evidence for the precision management and improvement of the efficacy of oral microbiota-mediated autoimmune diseases.

### The bilateral cross-talks between human microbiota and immune system

The microbiota plays a decisive role in the induction, training and functions of the host immune system. Early microbial exposure to infants is essential for regulating and influencing the formation of the immune system [20]. The bacterial components from the intestinal tract can be transported to the lactating mammary gland, which plays a crucial role in shaping the early-life gut microbiota and stimulating the immune system in infants [21]. *Lactobacilli* produce indole-3-aldehyde, and this microbial metabolite stimulates the production of IL-22 and IL17 by activating the aryl hydrocarbon receptor, contributing to the balance of the intestinal mucosal immune system [22]. Likewise, the symbiotic gut microbiota is beneficial for the maturation of gut-associated lymphoid tissues [23]. Severe inflammatory bowel disease (IBD) was observed in mice deficient in Peyer’s patches and lymph nodes [24]. Interestingly, retarded development of lymphoid follicles was observed in the small intestine of germ-free mice [25]. Similarly, germ-free or germ-depleted mice showed smaller secondary lymphoid tissue with lower cellularity [23]. These findings consistently indicate that the microbiota plays a vital role in the establishment and maintenance of the harmonious relationship between the host immune system and microbes, and abnormal changes in the microbiota composition might significantly affect the normal functions of the immune system, leading to immune-mediated diseases [26].

Conversely, the immune system also plays a fundamental role in establishing a sustainable and harmonious host-microbial relationship. The immune response to pathogenic microorganisms is the leading cause of host-mediated tissue damage, which might create a microenvironment that is beneficial for microbiota dysbiosis and invasion by pathogenic microbes. For example, the inflammatory response promotes the growth of aerobic bacteria and significantly decreases the survival of resident colonic bacteria, resulting in IBD [27]. Differences in the secretory function of Paneth cells also affect microbial composition. For instance, a significant distinction in intestinal microbiota composition was observed between human defensin 5 transgenic mice and wild-type mice [28].

### Microbiota dysbiosis and its driving factors

**Fig. 2:**
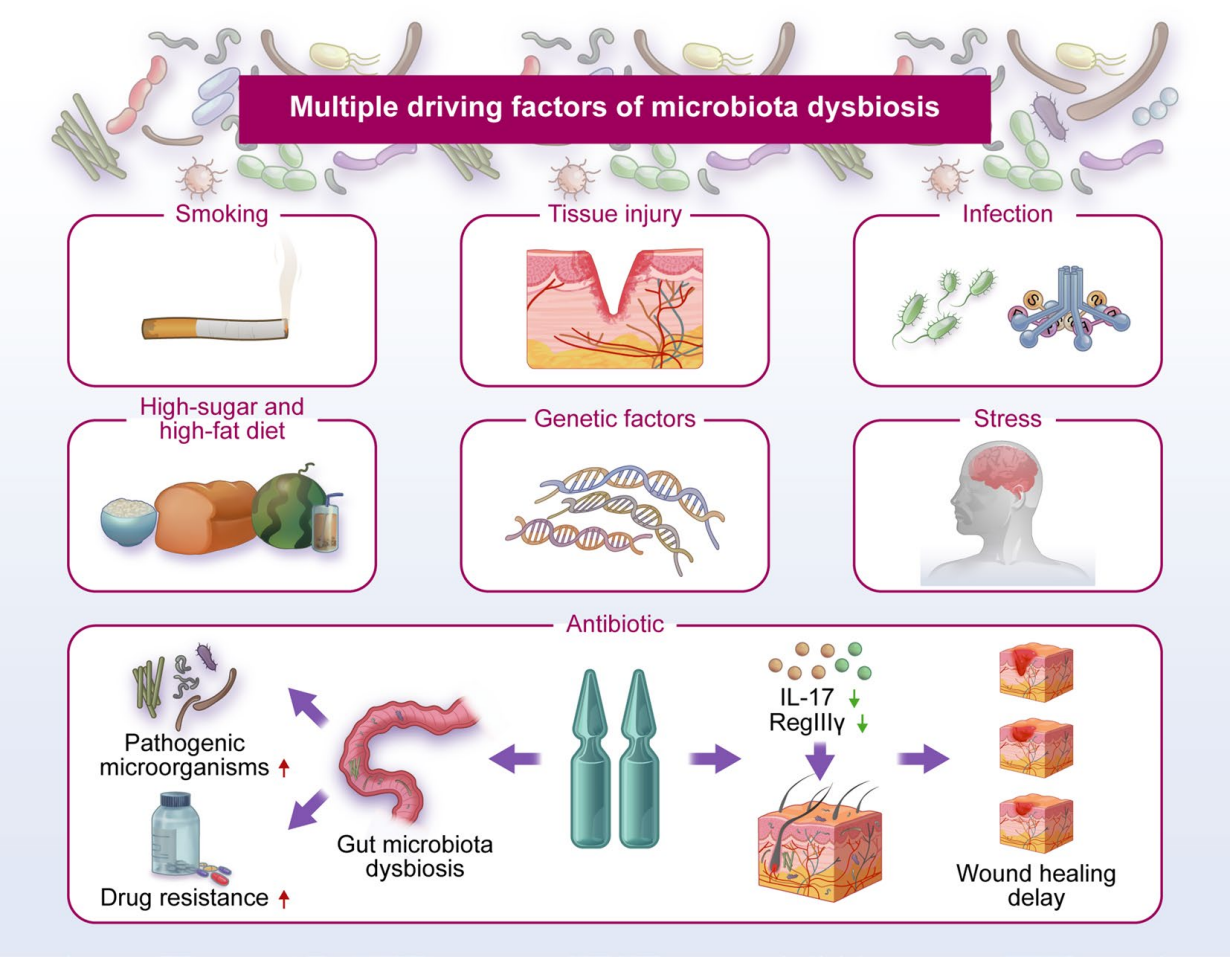
Multiple driving factors of microbiota dysbiosis. Genetic factors, dietary habits, stress, smoking, administration of antibiotics, tissue injury and infection could disrupt the abundance and composition of microbial communities

Microbiota dysbiosis, an imbalance in the composition and function of the microbiome, has a prominent impact on the initiation and progression of many systemic diseases, such as immunological disorders, cardiovascular diseases, diabetes, and respiratory diseases [29]. Dysbiosis is driven by many factors, such as host genetic factors, dietary habits, stress, smoking, administration of antibiotics, tissue injury and infection (Fig. 2) [3]. The increased consumption of carbohydrate-rich food and industrially processed flour and sugar are the two mainly dietary changes [30]. Dietary shifts contribute a significant role in shaping microbiota profiles [31]. A high-fat, high-sugar diet increased the release of dopamine, a neurotransmitter regulating pleasure, which recruit neural mechanisms driving food wanting [32, 33]. It is becoming increasingly clear that obesity is the risk factor for multiple autoimmune diseases, including RA, IBD and type 1 diabetes (T1D) [34]. It was reported that diet is a key factor in shifts of commensal balance as some oral pathogens were preferentially enriched in hunter-gatherers when compared with traditional farmers [35]. A diet high in fat and sugar can cause gut microbiota dysbiosis, leading to a variety of disorders such as obesity and non-alcoholic fatty liver disease. Sen et al. observed that rats treated with high-fat/high-sugar diet or low-fat/high-sugar diet exhibited an imbalance of gut microbiota with the characteristics of lower overall bacterial diversity and higher *Firmicutes*/*Bacteriodetes* ratio [36]. Moreover, *Bifidobacterium* spp. was important for maintaining gut/intestinal barrier function, and they were found to be absent in the gut microbiota of mice fed a high-fat diet [37]. Compared with high-fat diet, reduced level of plasma TNF-α and inhibition of systematic inflammation were found in the rats treated with low-fat diet [38]. Furthermore, the alterations of oral microbiota and recruitment of neutrophils were found in high-fat diet induced obesity mice model [39]. High-sugar diet may disturb the hepatic lipid metabolism by changing the structure of gut microbial community to cause hyperlipidemia and even non-alcoholic fatty liver disease [40]. Intake of probiotics, Grifola frondosa polysaccharides, healthy diet or polysaccharide peptides are proactive measures that contribute to the amelioration of microbiota dysbiosis caused by high-fat or high-sucrose diet [41–44]. Excessive administration of antibiotics disturbed the balance of the skin microbiota and retards wound healing by decreasing the expression of RegIIIγ and IL-17 [45]. In addition, overuse of antibiotics also disrupts the gut microbial balance, weakening the body's resistance to pathogenic microorganisms and increasing drug resistance [46]. The number and alpha diversity of gut microbiota in rats treated with antibiotics were significantly lower than the untreated groups [47]. Upon tissue injury, damaged cells release inflammatory chemical signals that facilitate the expansion of the pathogenic microbiota [48]. Moreover, it was reported that *P. gingivalis* induces microbial dysbiosis by affecting the normal function of the complement system [49]. The *P. gingivalis* activated Notch-1 signaling in oral epithelial cells to promote the expression of PLA2-IIA that was associated with oral dysbiosis [50].

### Microbiota dysbiosis and autoimmune diseases

To date, more than 80 autoimmune diseases has been identified, affecting a wide range of human body parts [51]. A healthy immune system effectively distinguishes self from non-self as well as harmless non-self from dangerous non-self [52]. Autoimmune diseases are characterized by immune response to self-antigens, including aberrant generation of autoantibody-producing B cells, autoreactive T cells, and proinflammatory cytokines [53]. The immune responses are induced by genetic and/or environmental factors acting on the immune system. The level of miR-146a was markedly elevated in THP-1 cells stimulated by IL-1β. The stimulated expression of miR-146a enhanced the tolerance of THP-1 cells to IL-1β, LPS, peptidoglycan, Pam and flagellin [54]. In addition, miR-146a might be involved in autoimmune diseases via the regulation of cytokines induced by 2D2 CD4 T cells. For example, increased IL-6 and IL-21 levels endowed the differentation of Th17 cells in 2D2 CD4 T cells in miR-146a–deficient mice, which induced more severe T-cell-mediated experimental autoimmune encephalomyelitis [55]. The production of inflammatory cytokines in *P.gingivalis* lipopolysaccharide-stimulated B cells was inhibited by miR-146a [56]. In addition, the expression of miRNA-146a-5p unregulated by Resolvin D1 depleted the levels of connective tissue growth factor, which might ameliorate the severity of RA [57, 58]. Dysbiosis of the oral microbiota (such as microbial diversity reduction and the high level of genus *Capnocytophaga*) has been found in patients with loss of STAT3 function [59]. Oral microbiota dyregulation has been increasingly considered as a potential risk factor for causing autoimmune diseases, even though the underlying mechanisms remain elusive. At the molecular level, molecular mimicry, autoantigen overproduction, and amplification of autoimmunity by cytokines represent the potential mechanisms for oral microbiota mediated autoimmune disorders. At the cellular level, the translocated oral microbiota directly interacts with immune and tissue cells and contributes to the pathogenesis of systemic autoimmunity [60]. Autoimmune diseases are typically defined by dyregulation of adapitive immune system. In contrast, autoinflammatory diseases result from an aberrant innate response, characterized by fewer T and B cells involvement or autoantibod production [61]. Polymorphonuclear neutrophils expressing major histocompatibility complex (MHC) class II antigens or the T-cell costimulatory molecules CD80 or CD86 in patients with chronic inflammatory diseases induced proliferation of T cells [62, 63]. Importantly, IL-1β, a robust pro-inflammatory cytokine, is secreted by macrophages and activate IL-1 receptor signaling to trigger the defferentiation of Th17 cells [64]. These innate immune cells including neutrophils and macrophages are critical in activating adaptive immune system by antigen presenting [65]. In addition, aberrant inflammatory response is closely correlated with the development autoimmune diseases, such IBD, RA and T1D [66, 67]. Collectively, the innate immune system is the first barrier against pathogen infection and tissue damage in host immune denfense, and subsequently palys a vital role in activating adaptive immunity [68].

Under harmonious host-microbial relationships, the microbiota provides vitamins, protects mucosal immunity and prevents colonization by pathogenic microorganisms, while the host offers an ecological niche and survival substances for the microbiota. Under dysbiosis, the mutually beneficial relationship between the host and microbes is disrupted, leaving the host more vulnerable to attacks by exogenous and endogenous pathogenic microorganisms. The host immune system in turn triggers a series of immune response processes, which might further aggravate microbiota dysbiosis.

Toll-like receptors (TLRs), which are germline-encoded pattern recognition receptors (PRRs), recognize pathogenic microorganisms and activate host signaling pathways to induce the production of cytokines and other inflammatory mediators [69]. In addition, TLRs promote the maturation of antigen-presenting cells (APCs), such as dendritic cells and macrophages, and induce the differentiation of T and B lymphocytes into effector cells, thereby regulating adaptive immunity [70–73]. Therefore, TLRs play an indispensable role in maintaining host-microbe homeostasis [74]. However, excessive activation of TLRs might lead to uncontrolled production of cytokines and inflammatory mediators, promoting the initiation and development of autoimmune diseases. The dying microbes in the dysfunctional flora release active substances such as lipids, nucleic acids, and proteins that induce the expression of TLRs, exacerbating the severity of autoimmune diseases [75]. Xu et al. found that *P. gingivalis* inhibited the host-protective TLR2-MyD88 pathway by proteasomal degradation of MyD88 and concurrently activated the host-detrimental TLR2-Mal-PI3K pathway, which destroyed immune function by blocking cellular phagocytosis [76].

Accumulating evidence has demonstrated that microbiota dysbiosis is the critical factor for engendering autoimmune diseases [77]. For instance, the in vivo results showed that intestinal microbial imbalance promoted the initiation of autoimmune pancreatitis by enhancing the production of IFN-α and IL-33 released by plasmacytoid dendritic cells [78]. In addition, intestinal microbiota dysbiosis caused by constipation increased the number of Th17 cells and Teff17 cells in the brain and spinal cord as well as promoted the secretion of cytokines in serum, which deteriorated experimental autoimmune encephalomyelitis in mice [79]. Collectively, gaining deep insights into the relationship between microbiota dysbiosis and autoimmune diseases is crucial for reducing the occurrence of autoimmune diseases and improving prognosis.

The oral cavity is exposed to the external environment and anatomically connected with the gastrointestinal tract. Thus, oral bacteria escaping the inhibition of stomach acid can spread through the gastrointestinal tract and are closely correlated with various systemic diseases, such as IBD and RA [80, 81]. Zhang et al. found that the oral *Campylobacter* species were detected in the intestinal tissues and stool samples [82]. A significant overlap between the fecal and oral microbiota has been reported, with approximately 45% similarity in bacterial taxa. These findings indicate that it is common for the ectopic colonization of oral bacteria in gastrointestinal tract [83]. Thus, the oral microbiota may play an important role in regulating the composition and structure of intestinal microbiota and have a great influence on the development of autoimmune diseases.

Administration of *P. gingivalis* significantly altered the gut microbiota in the mice. At the phylum level, *Deferribacteres* was increased and *Bacteroidetes* was decreased. Increased *Deferribacteriaceae*, *Gemellaceae*, and *Clostridiaceae* and decreased *Paraprevotellaceae* and *Mogibacteriaceae* were observed at the family level. Increased *Coriobacteriaceae*, *Gemellaceae*, and *Clostridiaceae* and decreased *Prevotellaceae*, *Mogibacteriaceae*, *Dorea*, *Butyricicoccus*, and *Bilophila* were found at the genus level [84]. The composition of the gut microbiota was disturbed in mice even receiving a single administration of *P. gingivalis*, with elevated levels of phylum *Bacteroidetes* and serum endotoxin and a decreased level of phylum *Firmicutes* [85]. It was reported that inflammation was promoted by gut microbiota dysbiosis via chemokine C–C chemokine ligand 5, which was important for recruiting lymphocytes in the intestine [86]. The endotoxemia induced chronic inflammation and insulin resistance, which contributed to the development of T1D [87]. Intestinal oxidative stress, barrier damage, inflammation and systemic autoimmune responses were common in MRL/lpr mice (a SLE animal model) with aberrant gut microbiome [88]. Oxidative stress promoted the development of autoimmune diseases including autoimmune hepatitis (AIH) and SLE by inducing apoptosis and inflammation [89]. Gut barrier damage in MRL/lpr mice was evidenced by increased levels of fecal albumin and IgA and a decreased level of gut tight junction protein (TJP) ZO-2 [88]. Also, intestinal permeability was increased due to downregulation of TJP-1 and occluding [85]. Thus, the gut microbiota dysbiosis leads to increased intestinal permeability, which in turn promotes the translocation of pathogens and other harmful microbial molecules to bloodstream and even distant organs. In addition, gut microbiota dysbiosis was observed in C57BL6/J mice administrated with salivary samples from periodontitis patients, with an increased abundance of *Porphyromonadaceae* and *Fusobacterium.* In addition, the levels of pro-inflammatory cytokines and chemokines such as IL-1β, IL-6 and colony-stimulating factor 1 were elevated, while the expression of anti-inflammatory cytokines such as IL-10 were decreased in colon tissue [90]. Apart from gut microbiota dysbiosis, the increase of TH17 cells proportions among mesenteric lymphocytes and IL-17 in sera were found in mice orally administration of *P. gingivalis*, followed by aggravation of collagen-induced arthritis [91]. *P. gingivalis* promotes the production of RA-related cytokines and activation of monocytes through TLR pathways [92]. Intestinal CD4 T cells and inflammation were indirectly induced due to the change in the gut microbiome by *P. gingivalis* [93]. The levels of TLR4 and CD14 were significantly higher in the livers of MRL/lpr mice [88]. Thus, oral bacteria promote gut microbiota dysbiosis by translocation and subsequently aggravating autoimmune diseases, which highlights the importance of the oral-gut-microbiota axis in the pathogenesis of autoimmune diseases [94].

### The major pathways by which oral microbiota dysbiosis affects autoimmune diseases

#### Microbial translocation

The main possible pathways by which oral pathogenic bacteria reach distant tissues and organs to trigger autoimmune diseases are the hematogenous and enteral routes. Invasive oral treatments, oral mucosal ulcers, injuries, and periodontitis provide convenient entry points for oral pathogenic microorganisms to invade the circulatory system. Subsequently, the oral bacteria and their soluble bacterial products can reach distant organs via the bloodstream [95, 96]. For example, a mouse model of dextran sodium sulfate (DSS)-induced colitis was used to investigate the relationship between *Streptococcus mutans* and colitis. The results demonstrated that *S. mutans* may increase the severity of colitis, as it promoted the secretion of IFN-γ when entering the bloodstream [97]. In addition, in a murine model of colorectal cancer, intravenously injected *Fusobacterium nucleatum* colonized tumor tissues [98]. In addition, the bacteria detected in the oral cavity of the mouse periodontitis model were also found in isolated liver and spleen cells in vitro [99]. Due to the hematogenous dissemination of *P. gingivalis*, more severe cartilage destruction and higher expression of IL-17 in joints were found in mice orally treated with a mixture of *P. gingivalis, T. denticola*, and *Tannerella forsythia* [100].

In addition to the hematogenous route, the enteral route is a major route for oral bacterial dissemination. Increased intestinal permeability is associated with the pathogenesis of many autoimmune diseases, such as type I diabetes, multiple sclerosis (MS), and SLE [101]. An enormous number of oral resident bacteria are swallowed into the digestive tract per day, accompanied by 1.5 L of saliva [102]. Oral microorganisms are ordinarily damaged by stomach acids, so they fail to pass through the stomach to the gut. However, some oral pathogens, such as *P. gingivalis*, have the ability to withstand stomach acidity and colonize the intestine [80]. The gut permeability and the level of blood endotoxin were significantly increased in mice that were orally administered *P. gingivalis* [103]. Generally, a healthy gut microbiota or gut barrier can confer resistance to ectopic colonization by oral bacteria. However, bacteria from the oral cavity can easily invade the gut when the intestinal microbiota is disrupted due to the administration of antibiotics. *Klebsiella* strains, as oral-derived intestinal pathogenic bacteria, possess the ability to resist a variety of antibiotics, including ampicillin (Amp) and tylosin (Tyl). For instance, compared with antibiotic-naïve mice, mice treated with Amp or Ty1 had a perturbed gut microbial profile, which was beneficial for colonization by Kp-2H7. *Klebsiella* strains strongly induce the accumulation of intestinal TH1 cells, leading to exacerbation of inflammatory diseases through abnormal activation of the intestinal immune system [104]. To investigate the effect of oral microorganisms on intestinal epithelial barrier dysfunction, Nattramilarasu et al. cultured colonic epithelial cell monolayers (HT-29/B6-GR/MR) and M1-macrophage-like THP-1 cells together in vitro. After infection with *Campylobacter concisus,* THP-1 cells promoted the release of cytokines (TNF-α, IL-1β, and IL-6) and consequently increased the permeability of the intestinal epithelium [105]. Oral pathobionts such as *Klebsiella* and *Enterobacter* derived from ligature-induced periodontitis and DSS-treated mice stimulated inflammasome production in colonic mononuclear phagocytes, followed by increased inflammation in the colonic mucosa. Additionally, *Klebsiella* and *Enterobacter* activated oral pathobiont-reactive Th17 cells, which had a tendency to migrate toward the inflamed gut [106]. Thus, colonization by oral-associated pathogens in the intestinal system through the enteral route plays a crucial role in the exacerbation of autoimmune diseases [6].

#### Molecular mimicry

“Molecular mimicry” was coined by R.Damian in 1964. It is demonstrated that more than 30 viral proteomes, especially the proteomes of human T-lymphotropic virus 1, Rubella virus, and hepatitis C virus, have a substantial pentapeptide overlap with the human proteome [107]. These observations strongly confirmed the pathogenesis of immune diseases might be due to the sharing of amino acid sequences between microbiome and the host. As a rich source of antigen, the oral cavity harbors one of the most diverse and abundant microbial communities. Molecular mimicry is involved in oral microbiota-mediated chronic infection in localized and remote organ autoimmunity. Periodontopathic bacteria (*P.gingivalis* and *Aa*) express the GroEL-like protein, which is a form of HSP60 family [108, 109]. Of note, higher levels of antibodies against human HSP60 were also observed in the sera of periodontitis patients [110]. It was reported that *Streptococcal* M protein exhibits structural similarities with cardiac myosin, which underlies a potential pathogenic mechanism of chronic rheumatic heart disease [111]. Increasing evidence has shown that dysregulation of the oral microbiome induces autoimmune diseases through molecular mimicry [112]. For instance, the epitopes of *P. denticola *and *Bacillus cereus* have a high degree of similarity with the self-autoantigen Ro 60KDa, which is often detected in SLE and Sjögren’s syndrome (SS). In addition, *P. gingivalis, Streptococcus* spp*.,* and *F. nucleatum* have similar epitopes as the self-autoantigen pyruvate dehydrogenase complex E2 (PDC-E2), which is the possible mechanism underlying the initiation and progression of primary biliary cirrhosis (PBC) [113].

#### Autoantigen overproduction

Cytokines regulate some proteolytic enzymes to hydrolyze extracellular proteins, generating remnant epitopes. The remnant epitopes serve as autoantigens [114]. Immune cells recognize autoantigens and produce autoantibodies. Thus, remnant epitopes are the key factors for the pathogenesis of autoimmune diseases [115]. Neutrophils were recruited and activated by IL-8/CXCL8 to secrete collagenase/ matrix metalloproteinases-8 (MMP-8) and gelatinase B/MMP-9. Gelatinase B in turn potentiated IL-8 to enhance activation of neutrophils and then increase the number of neutrophils and lymphocytes in the joint. Cartilage collagen type II was degraded to multiple protein peptides by MMP-8, which subsequently induced the activation and proliferation of autoimmune T cells [116]. Autoantibodies produced by nonspecific activation of low-affinity autoreactive B cells were triggered by infection. Soulas et al. showed that the levels of rheumatoid factors were higher in Tg mice infected by *Borrelia burgdorferi* [117]. Of note, the microbiota produces large amounts of proteolytic enzymes, contributing to autoantigen overproduction. For instance, the supernatants of *P. gingivalis* effectively cleave fibrinogen, fibronectin and type I collagen, which may promote the production of autoantibodies [118]. In addition to remnant epitopes, enzymatic antigen modification is critical in the generation of autoantigens. For instance, *P. gingivalis* gives rise to the production of bacterial PADs, which catalyzes the citrullination of proteins [119]. Proteins harboring citrullinated epitopes can be recognized by anti-citrullinated protein antibodies (ACPAs), which is the common pathological mechanism of RA [120, 121]. Furthermore, *Aa* produces pore-forming toxin LtxA to induce neutrophil hypercitrullination in patients with chronic periodontitis, and citrullinated autoantigens are the main immune targets in RA [18].

#### Amplification of autoimmunity by cytokines

**Fig. 3:**
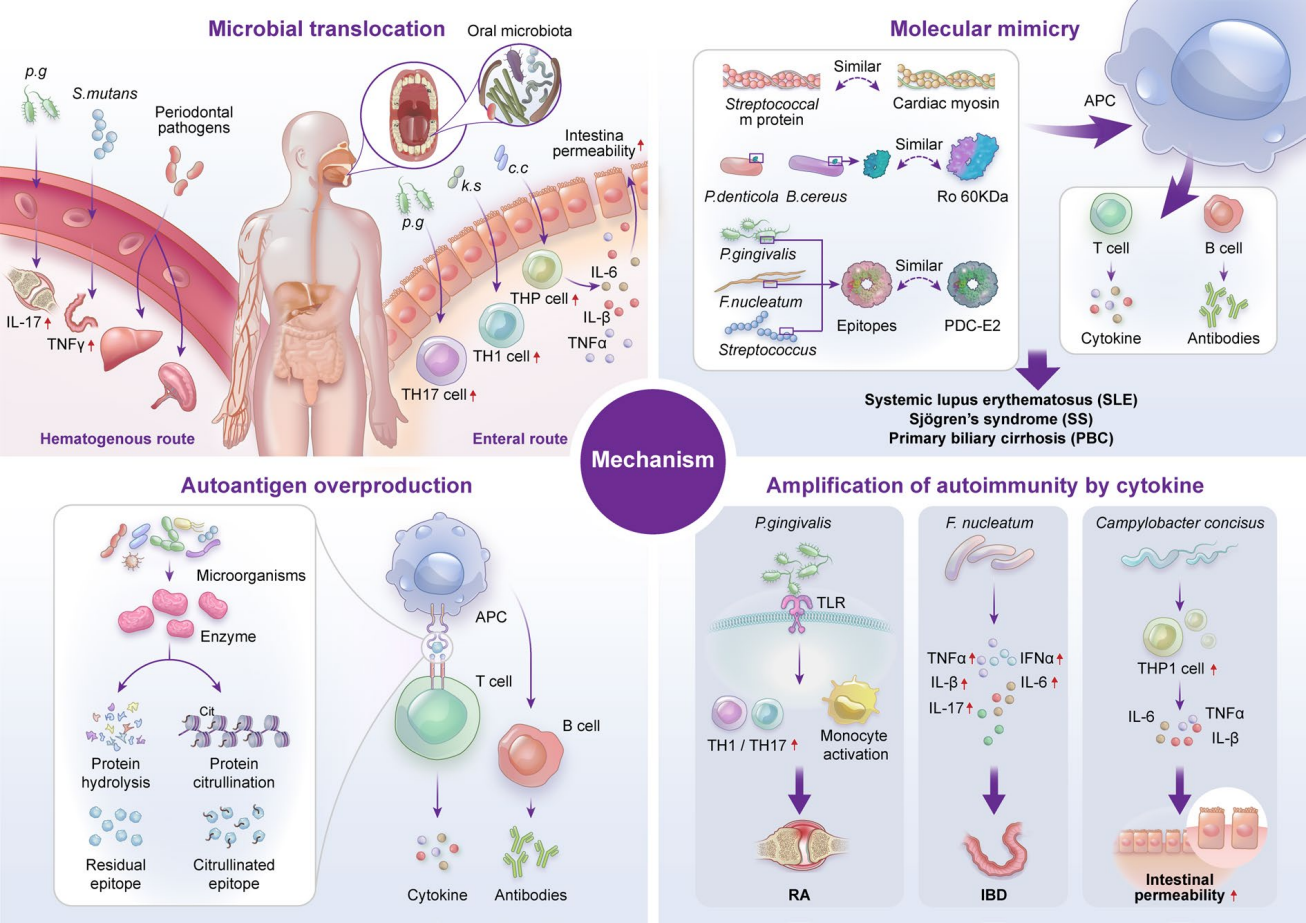
The critical mechanism of oral microorganisms in autoimmune diseases. The major pathways of oral microorganisms-autoimmune diseases axis are shown among the microbiota translocation, molecular mimicry, autoantigen overproduction and amplification of autoimmunity by cytokine. Top left: communication between oral microbiota and the host involves the hematogenous and enteral routes. Under the adverse conditions such Ulcers and periodontal destruction, oral microbiome, such as *P.g*, *S.mutans*, and other periodontal pathogens arrive at the distant organs, subsequently increasing the release of cytokines and affecting intestinal barrier integrity. Change in the oral microbiota composition can also increase the response of molecular mimicry, autoantigen overproduction and amplification of autoimmunity by cytokine. Top right: the antigen epitopes of *P. denticola*, *B. cereus*, *P.gingivalis*, *F.nucleatum*, and *Streptococcus* increase. Bottom left: The production of residual and citrullinated epitope increase due to the hydrolysis of enzymes form microorganisms. The epitopes might be preferentially captured by APC which presented antigen epitopes to T cells, leading to the production of inflammatory factors. Subsequently, B cell response is activated to produce antibodies. Bottom right: Pathogenic microorganisms including *P. gingivalis*, *F. nucleatum* and *C. concisus* promote the release of TH1/TH17, TNFα, IFNα, Ilβ, IL-6, IL-17. *p.g*, *P.gingivalis*; *k.s*, *Klebsiella* strains; *c.c*, *C. concisus*; APC, Antigen presenting cell; TLR, Toll-like receptor; RA, Rheumatoid arthritis; IBD, Inflammatory bowel disease; Ro60, Anti-dsDNA, Anti-double stranded DNA antibody; Ro60, Ro ribonucleoprotein 60 KDa; PDC-E2, Pyruvate dehydrogenase complex E2

A large body of evidence has revealed that cytokines are actively involved in the initiation and progression of autoimmune diseases [122]. Thus, the proinflammatory microenvironment modulated by the microorganisms is correlated with autoimmune responses [123]. Infection of microorganisms promoted the apoptosis of intestinal epithelial cells, and subsequently elicited the presentation of self-antigens by MHC-II molecules and the generation of autoreactive Th17 cells, leading to the occurrence of auto-inflammation and the production of autoantibodies [124]. It was reported that the level of IL-17 was significantly increased in RA, MS, and IBD [125, 126]. *P. gingivalis* promotes the production of RA-related cytokines and monocyte activation through TLR pathways. Similarly,* F*. nucleatum enhances the expression of TNF-α, IFN-gamma, IL-1β, IL-6, and IL-17, which further exacerbates IBD [127]. In addition, *C. concisus* promotes the secretion of proinflammatory cytokines from THP-1 cells, such as TNF-α, IL-1β, and IL-6, which increase the antigen permeability of the colonic epithelial barrier [105]. It should be pointed out that periodontitis fosters a proinflammatory microenvironment, and the levels of IL-1β, IL-4, IL-10, and IL-17A were significantly higher in diseased sites than healthy sites in the same oral cavity from periodontitis patients [128]. IL-1β stimulated T cells to promote autoimmunity by increasing the production of IL-17 [129]. Amplification of Th17 cells and the production of their effector cytokines (IL-17A, IL-17F, IL-21, CCL20) can be blocked by intravenous immunoglobulin therapy [130]. The major pathways by which oral microbiota dysbiosis affects autoimmune diseases have been summarized in Fig. 3. In addition, the effects of oral microbiota on autoimmune diseases have been summarized in Table 1.

**Table 1:** The effects of oral microbiota on autoimmune diseases

Autoimmune disease	Pathogenic Oral microbiota	Key mediators	Promoter or suppressor	Mechanism	References
IBD	*Streptococcus mutans*	IFN-γ	Promoter	Microbial translocation (hematogenous route)	[97]
IBD	*Klebsiella* strains	TH1 cells	Promoter	Microbial translocation (enteral route) and amplification of autoimmunity by cytokines	[104]
IBD	*F. nucleatum*	TNF-α, IFN-γ, IL-1β, IL-6, and IL-17	Promoter	Amplification of autoimmunity by cytokines	[127]
IBD	*C. concisus*	THP-1 cells	Promoter	Amplification of autoimmunity by cytokines	[105]
	*P. gingivalis*	PADs	Promoter	Autoantigen overproduction	[119]
RA	*Aggregatibacter actinomycetemcomitans*	ACPA	Promoter	Autoantigen overproduction	[18]
RA	*P. gingivalis*	IL-17	Promoter	Microbial translocation (hematogenous route)	[100]
RA	*P. gingivalis*	TLR	Promoter	Amplification of autoimmunity by cytokines	[92]
SLE	*Aggregatibacter actinomycetemcomitans, P. gingivalis, T. denticola, and C. ochracea*	Anti-dsDNA antibodies	Promoter	Autoantigen overproduction	[19]
SLE、SS	*P.denticola* and *B.cereus*	Ro60	Promoter	Molecular mimicry	[113]
PBC	*P.gingivalis, Streptococcus spp.,* and* F.nucleatum*	PDC-E2	Promoter	Molecular mimicry	[113]

*IBD* Inflammatory bowel disease, *RA* Rheumatoid arthritis, *SLE* systemic lupus erythematosus, *SS* sjogren syndrome, *PBC* primary biliary cholangitis; *PAD*s peptidylarginine deiminases, *ACPA* anti-citrullinated protein antibodies, *TRL* toll-like receptor, *PDC-E2* Pyruvate dehydrogenase complex E2; *Anti-dsDNA* anti-double stranded DNA antibody; *Ro60* Ro ribonucleoprotein 60 KDa

### The relationship between the oral microbiota and autoimmune disease

#### The oral microbiota and IBD

Ulcerative colitis (UC) and Crohn's disease (CD) represent two common types of IBD with chronic and recurrent characteristics [131]. Genetic predisposition, environmental exposure, microbiota dysbiosis and ethnic background are the major contributors to the initiation and progression of IBD [132].

Recently, there has been growing evidence that dysbiosis of the oral microbiome plays a key role in triggering and exacerbating IBDs. A significant increase in the abundance of *Saccharibacteria* (TM7), *Absconditabacteria* (SR1), *Leptotrichia, Prevotella, Bulleidia*, and *Atopobium* was found in saliva specimens from patients with IBD [133]. *Streptococcus* and *Enterobacteriaceae* species were enriched, while *Lachnospiraceae* and *Prevotella* were depleted, in saliva samples from patients with UC. *Veillonellaceae* was abundant, while significant depletion of *Neisseriaceae* and *Haemophilus* were observed, in saliva samples from patients with CD [134]. Differences in the sample size and sequencing technologies might be the reasons for the contradictory findings regarding the level of *Prevotella* in the above two studies. The salivary microbiota profile of CD patients with periodontitis was significantly different from that of those with periodontitis alone and healthy individuals, characterized by an increase in the abundance of *Proteobacteria* and *Firmicutes* [135]. It is well established that increased abundance of *Proteobacteria* often leads to an imbalanced gut microbiota [136]. In addition, approximately 30 abundant bacterial taxa, including *g.Prevotella, f.Prevotellaceae,* and *p.Bacteroidetes,* were more abundant in saliva samples from patients with active CD than in samples from control subjects or those in the remission phase [137]. *Streptococcus salivarius* was consistently detected in saliva and fecal samples from patients with CD by whole-genome shotgun sequencing, which provides compelling evidence for the occurrence of ectopic gut colonization [138, 139]. It should be noted that the majority of *Campylobacter* species detected in the intestinal tissues and stool samples from patients with IBD were human oral *Campylobacter* species [82]. The detection rate of Campylobacter species, especially* C. concisus*, is significantly higher in colonic biopsy samples of UC patients than in control samples [140]. In general, *C. concisus* is rarely observed in healthy intestines [141]. The pathogenicity of *C. concisus* in the intestine manifests as destruction of the colonic epithelial barrier, which results in the enhancement of antigen permeability and further facilitates the damage caused by other enteric bacterial species [105, 142].

Mechanistically, the salivary microbiota from patients with periodontitis not only significantly changes the structure of gut microbes but also exacerbates colitis by reducing the level of unsaturated fatty acids and enhancing arachidonic acid metabolism [143]. *Klebsiella* strains isolated from the salivary microbiota with multidrug resistance were capable of colonizing the colon and increasing the numbers of proinflammatory TH1 cells, which subsequently aggravated the progression of IBD [104]. In addition, PADs, an enzyme or virulence factor specific to *P. gingivalis*, promotes the production of Th17 cells and IL-17, causing aggravation of UC [144]. It has been demonstrated that *P. gingivalis* dramatically increases the severity of colitis by breaking down TJPs to increase the permeability of the intestinal epithelium [145]. *F. nucleatum* is one of the resident bacteria and opportunistic pathogens in the human oral cavity [83, 146, 147]. It is closely associated with IBD progression, acting by damaging intestinal epithelial integrity and increasing intestinal mucosa permeability [127]. More severe epithelial cell damage was found in mice simultaneously treated with DSS and *F. nucleatum* [148]. Su et al. revealed that *F. nucleatum* exacerbated UC by promoting intestinal epithelial cell autophagy. In addition, *F. nucleatum* activates both the endoplasmic reticulum stress and IL-17F/NF-kappaB pathways, leading to the destruction of the intestinal mucosal barrier [149, 150]. Moreover, *F. nucleatum* regulates proinflammatory M1 macrophages via the AKT2 signaling pathway to exacerbate UC [151].

#### The oral microbiota and RA

RA is a chronic autoimmune disease with a complex multifactorial etiology. The typical clinical manifestations of RA are symmetrical inflammation and pain in the small joints of the hands and feet. The concordance rate of RA in twins is extremely low, indicating the significance of environmental risk factors for the initiation and development of RA [152].

**Fig. 4:**
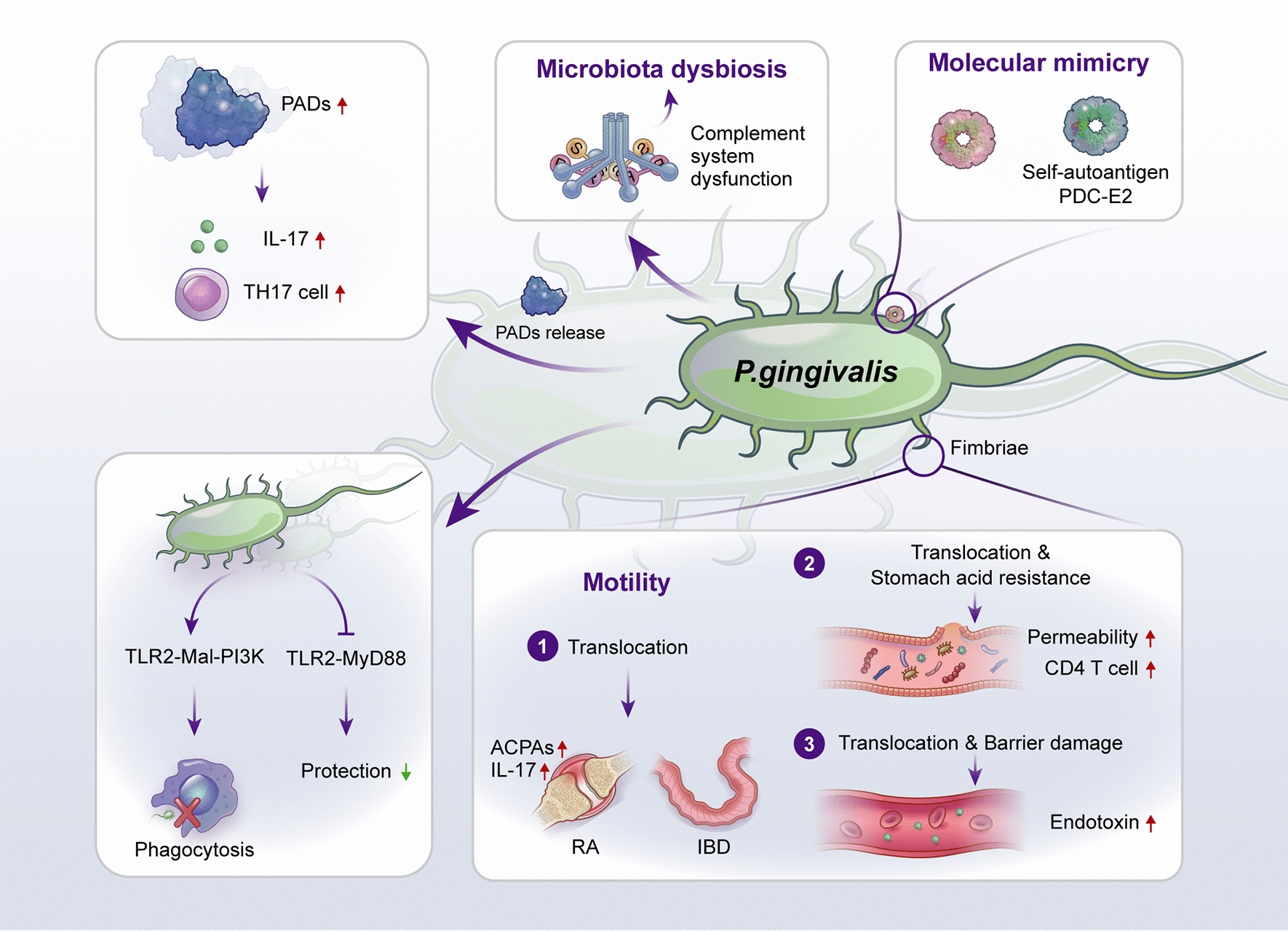
Graphical description of targeting the possible mechanisms of *P. gingivalis* for triggering autoimmune diseases. *P. gingivalis* is a Gram-negative anaerobic bacillus, mainly locating in the oral cavity. Bottom right: However, due to barrier damage and the function of withstanding stomach acidity, distant translocation of *P. gingivalis* into joints and enteral tissue by hematogenous dissemination and enteral route has been found in animal models. After successful colonization and survival, the higher level of endotoxin in blood and IL-17 as well as ACPAs in joints could be found in mice treated with *P. gingivalis* or mixed oral pathogenic microorganisms containing *P. gingivalis*. Bottom left: Moreover, *P. gingivalis* possesses immunosuppressive activities by activating the host-detrimental TLR2-Mal-PI3K pathway to blocked cellular phagocytosis. Also, *P. gingivalis* inhibited the host-protective TLR2-MyD88, which may further weaken the protective function of the immune system. Top middle: In addition, affecting the normal function of the complement system by *P. gingivalis* conducive to oral microbiota. Top left: PADs from *P. gingivalis* could catalyze the citrullination of proteins, which promotes the production of Th17 cells as well as IL-17 and exacerbates UC. Top right: *P. gingivalis* have similar epitopes as the self-autoantigen PDC-E2, which is the possible mechanism underlying the initiation and progression of primary biliary cirrhosis. ACPAs, anti-citrullinated protein antibodies; PADs, peptidylarginine deiminases; PDC-E2, pyruvate dehydrogenase complex E2

It has been demonstrated that *P. gingivalis* is closely correlated with the occurrence and development of RA, and the detailed molecular mechanisms of *P. gingivalis* for triggering and promoting autoimmune diseases have been summarized in Fig. 4. The correlation analysis revealed that the prevalence of *P. gingivalis* was significantly higher in tongue biofilms from non-remission RA patients than in those from remission patients [153]. The expression of *P. gingivalis* was significantly increased in individuals positive for anti-cyclic citrullinated peptide compared with controls [154]. *P. gingivalis* triggered the initiation of periodontitis and joint inflammation in rats [155]. In addition, collagen-induced arthritis mice injected with *P. gingivalis* developed more severe synovial inflammation and bone destruction in joints [156]. Similarly, collagen-induced arthritis mice that were orally administered *P. gingivalis* suffered from more severe arthritis. Interestingly, *P. gingivalis* affects the progression of RA by directly migrating into joints and inducing the secretion of ACPAs. The fimbriae function of *P. gingivalis* and the assistance of dendritic cells, macrophages, and neutrophils might contribute to its migration. collagen-induced arthritis mice injected with anti-FimA antibody had less severe arthritis, as anti-FimA antibody inhibited the attachment and aggregation of *P. gingivalis* [157]. A reduction in periodontal inflammation and anti-*P. gingivalis* IgG levels was observed in patients with PD after periodontal treatment. However, inflammation and the level of ACPAs changed little in the joint due to ectopic colonization by *P. gingivalis* [158]. The development of RA is also affected by *streptococcal* species. Moentadj et al. found that Streptococcus species were abundant in the oral cavity of RA patients. Following the administration of *streptococcal* cell walls (SCWs) from *Streptococcus. parasalivarius* strains, ZAP-70-mutant SKG mice developed chronic arthritis [159]. A case‒control study reported that *Treponema* was greatly enriched in the PD and RA groups compared with the healthy controls by analyzing subgingival plaques [160]. In addition, early-stage RA patients and at-risk individuals had higher levels of *Prevotella* and *Veillonella* in saliva samples [161]. Moreover, the levels of *Streptococcus parasanguinis* and *Actinomyces meyeri* were higher in subgingival plaque samples of RA patients than in non-RA individuals [162]. The currently available animal models for dissecting the interactions between oral microbiota dysbiosis and autoimmune diseases (IBD and RA) are summarized in Table 2.

**Table 2:** The currently available animal models for dissecting the interactions between oral microbiota dysbiosis and autoimmune diseases

Mice	Disease model	Experimental intervention	Pathological changes	Consequence	References
B10.RIII	Collagen-induced arthritis	Oral administration of *P. gingivalis*, *T. denticola* and *T. forsythia*	Increased periodontal bacteria in synovial joints	Exacerbations of RA	[100]
C57BL/6N	Experimental periodontitis	Oral administration of *P.gingivalis*	Increased gut permeability and blood endotoxin levels	Increased risk of systemic diseases	[103]
Il10 WT B6	NA NA	Oral administration of Amp and Kp-2H7 Oral administration of saliva from patients with active UC	Accumulation of TH1 cells in colonic tissues	Exacerbations of colitis	[104]
C57BL/6	DSS-induced colitis	Ligature-induced periodontitis	Increased inflammasome production in colonic mononuclear phagocytes	Exacerbations of intestinal mucositis	[106]
C57BL/6	DSS-induced colitis	Oral administration of *F. nucleatum*	Increased damage to epithelial cells	Exacerbations of UC	[148]
DBA/1 J	Collagen-induced arthritis	Injection with *P. gingivalis*	Severe synovial inflammation and bone destruction in joints	Exacerbations of RA	[156]
DBA1/J	Collagen-induced arthritis	Injection with anti-FimA antibody	Inhibiting the attachment and aggregation of *P.gingivalis*	Alleviation of RA	[157]
BALB/c	SKG model of arthritis	Administration of SCWs from *S. parasalivarius* strains	Enrichment of SCW in synovial tissue	Induction of chronic arthritis	[159]

*UC* ulcerative colitis, *DSS* dextran sulfate sodium, *SCW* Streptococcal cell walls, SKG, BALB/c ZAP-70W163C-mutant. Kp-2H7, *Klebsiella pneumoniae* 2H7

#### The oral microbiota and SLE

SLE is a type of chronic autoimmune connective-tissue disease of unknown etiology that affects multiple organs and leads to tissue damage and extensive inflammation. SLE is more common in women, with a global widespread prevalence of 0.02–0.24% [163]. Notably, genetic makeup, environment, sex, hormones, and the hypothalamic‒pituitary‒adrenal axis play essential roles in increasing the susceptibility to SLE [164].

Recently, a growing body of evidence has indicated the close correlation between the oral microbiota and SLE [165, 166]. Pessoa et al. revealed that 14 unique subgingival bacteria were markedly enriched in SLE patients compared to controls. In addition, the abundances of *T. denticola* and *T. forsythia* were increased in SLE-active periodontal sites compared with SLE-inactive sites and sites in healthy controls [167]. The microbial diversity was increased in the saliva samples from SLE patients, as shown by 16S ribosomal RNA gene sequencing. *Veillonella*, *Streptococcus*, and *Prevotella* were the predominant bacteria detected in saliva samples [168]. However, Li et al. discovered that the oral microbiota diversity from mucosal swabs was reduced in SLE patients. The levels of *Sphingomonadaceae*, *Halomonadaceae*, and *Xanthomonadaceae* were dramatically decreased in SLE patients [12]. The inconsistent microbial diversity may be due to the differences in the number of samples.

Notably, periodontitis might activate immune responses by maintaining high expression of TLR2 and TLR4, resulting in aggravation of the occurrence and progression of SLE. Potential cross-recognition was observed between human self Ro60 and homologous peptides from the oral microbiota, which illustrated the potential ability of bacterial antigens to induce an autoimmune response [169]. The expression of TLRs was significantly reduced after periodontal treatments, and the symptoms of SLE were subsequently improved due to the inhibition of immune responses [170]. Additionally, higher levels of IL-6, IL-17A and IL-33 were detected in the saliva of SLE patients with chronic periodontitis. These inflammatory factors contribute to the progression of SLE by inducing the recruitment, activation and differentiation of immune cells to increase the autoimmune response and promote tissue injury [171–173]. Interestingly, the abundance of the beneficial bacteria of *Bacteroides* was negatively linked to SLE disease activity, indicating that manipulating the composition of the oral microbiota might be an effective strategy for treating SLE [168].

#### The oral microbiota and T1D

T1D is a T-cell mediated autoimmune disease characterized by insulin deficiency due to the destruction of pancreatic β-cells. T1D is the most common type of diabetes in children and adolescence [174]. Accumulating evidence has demonstrated that genetic predisposition and environmental factors (dietary factors, infections, air pollution and microbial composition) affect the pathogenesis of T1D [175–178]. Lower microbial diversity and higher abundance of opportunistic pathogens were found in the saliva samples from children with new-onset T1D compared to those detected in the saliva samples from healthy children or children with T1D in the chronic phase receiving insulin treatment [179]. The Phyla *Actinobacteria* and *Firmicutes*, including *Streptococcus* spp., *Actinomyces* spp. and *Rothia* spp were the predominant bacteria detected in oral swabs of T1D patients [180]. In addition, Moskovitz et al. revealed that *Streptococcus* was increased in the unstimulated saliva samples from T1D patients by using 16S rRNA sequencing [181]. *Aggregatibacter* was found only in patients with T1D, while it was absent in the healthy controls and patients with periodontal diseases [182]. *Capnocytophaga sputigena* and *C. ochracea* were the more common detected bacterial in subgingival samples from patients with T1D [183]. Noteworthy, a bidirectional relationship was found between periodontitis and T1D. Poor glycemic control deteriorates periodontal disease by aggravating gingival inflammation, increasing probing pocket depth, and accumulating dental plaque. In addition, glycosylated hemoglobin (HbA1c) was positively correlated with periodontal disease index [11, 184]. Importantly, the levels of HbA1c and prostaglandin E2 were significantly reduced in T1D patients after periodontal treatment [185].

Microbial communities residing in oral and intestinal niches may synergistically promote the progression of T1D, highlighting the important role of oral-gut microbiota axis. Metaproteomics revealed that the microbial taxa (*Alistipes* and *Faecalibacterium prausnitzii*) contributed to maintain the function of the mucous barrier, microvilli adhesion, and exocrine pancreas were depleted in new-onset T1D [186]. The gut microbial composition in the mice administered with salivary microbiota from a periodontitis patient was significantly different from that in health-associated oral microbiota-administered (HAO) mice. *Bifidobacterium, Peptostreptococcus, Atopobium, Mannheimia, Campylobacter, Olsenella, Lactobacillus,* and *Acidpropionibacterium* were abundant in periodontitis-associated microbiota-administered (PAO) mice, while *Cutibacterium, Streptococcus, Actinomyces, Faecalibaculum, Granulicatella, Gemella, Alloprevotella,* and *Staphylococcus* were enriched in HAO mice [187]. In addition, oral bacteria led to dysbiosis of intestinal microbiota. Elevated levels of *Bacteroides* and *Staphylococcus* and reduced level of *Lactobacillus* spp may result in a decrease in Th17 cells and an increase in the M1/M2 macrophage ratio [188]. *Bacteroides fragilis* (BF) tends to aggravate T1D under the condition of increased gut permeability [189]. With the assistance of oral microorganisms such as *P. gingivalis* or *F. nucleatum* that increase the intestinal mucosa permeability, BF-like gut microorganisms promote the progression of T1D [127, 145]. Importantly, oral microbiota changes the systemic metabolism by modulating gut microbiota. A higher abundance of 2-hydroxyisobutyric acid and hydroxybenzoic acid was found in PAO mice than in HAO mice [187]. Interestingly, patients with autoimmune diseases had a higher level of 2-hydroxyisobutyric acid and hydroxybenzoic acid [190].

#### The oral microbiota and SS

SS, including primary SS and secondary SS, is a chronic autoimmune disease. It is mainly characterized by oral and ocular dryness due to damage to salivary and lacrimal glands with the infiltration of lymphocytes [191]. SS is a heterogeneous and multifactorial disease and is typically more common in middle-aged and older women than in men [192]. A recent study showed that *Prevotella melaninogenica* from the mouthwash samples of SS patients could invade ductal cells. Meanwhile, it increased the expression levels of MHC molecules, including CD80 and IFN-γ [193]. The pathogenesis of SS may be interpreted by the microbial polypeptide activation hypothesis. Peptides from the oral microbiota activated Sjogren’s syndrome antigen A /Ro60- reactive T cells, which induced autoimmune responses in SS. In particular, one of the most effective activators was peptide-P106, which is from von Willebrand factor type A domain protein (vWFA) of *C. ochracea* [194]. Compared with the healthy controls, both pSS patients and non-SS sicca patients exhibited a perturbed salivary microbiome profile. In addition, significant differences in the salivary microbiota were found between pSS and non-SS sicca patients [195]. It is worth noting that there is no statistically significant difference in the main oral microorganisms between xerostomia patients and the normal population [196].

An increase in *Bacteroidetes* and *Actinobacteria* abundance and a decrease in *Proteobacteria* abundance were found in saliva samples of pSS patients by high-throughput sequencing [197]. *Veillonella* was approximately four times more abundant in saliva samples from patients with pSS than in healthy subjects [198]. Compared to healthy controls, a significant increase in the relative abundance of *Bifidobacterium, Dialister,* and *Lactobacillus* was observed in the salivary samples of pSS patients, while the expression of *Leptotrichia* was markedly decreased [199]. In the supragingival samples of SS, the abundance of *Veillonella parvula*, *F. nucleatum, ss vincenti*, and *Propionibacterium acnes* was significantly increased. In particular, the level of *V. parvula* was higher in subgingival and supragingival samples from SS patients than in healthy controls [200]. In tongue microbiome samples, De Paiva et al. discovered that the relative abundance of *Streptococcus* was higher in mice with SS, while that of *Leptotrichia*, *Fusobacterium, Bergeyella, Peptococcus,* and *Butyrivibrio* was decreased [201]. A lower oral microbial richness was observed in a mouse model of SS [201]. However, no significant differences in bacterial richness and diversity were found between pSS patients and healthy controls [197]. The inconsistent results for oral microbiota richness in SS need further evaluation.

#### The oral microbiota and ankylosing spondylitis (AS)

AS, a common form of axial spondyloarthritis, is an immune-mediated inflammatory spondyloarthropathy characterized by inflammation of the sacroiliac joints and spinal attachment points. There may be no clinical symptoms in the early stage, but bone fibrosis and calcification appear in the advanced stages of AS. To date, it is generally accepted that AS is influenced by multiple factors, including genetic background, immune reaction, and microbial infection. The exact pathogenic mechanism of AS has not yet been established [202, 203]. Arthritis was developed in the rats under the conventional feeding condition but not in those maintained in the sterile condition. In addition, intramural injection of the bacterial cell wall polymers resulted in peripheral arthritis and the inflammatory responses were blocked by the addition of metronidazole [204]. Wen et al. compared the gut microbial composition in AS patients and healthy controls with quantitative metagenomics analysis. An increased abundance of *P. melaninogenica, Prevotella copri,* and *Prevotella *sp. C561 and a decreased level of *Bacteroides* spp were found in AS patients [205]. An unbalanced gut microbiota was observed in patients with AS, showing decreased quantity of bacteria and a significant increase in *Bifidobacterium* species [206]. Notably, the levels of *Veillonellaceae*, *Brucella* spp., and *C. concisus* were significantly higher in saliva samples from patients with AS, while the abundance of *Streptococcus* was decreased [207]. Stoll et al. reported that the levels of salivary *Rothia mucilaginosa* and plaque-derived *Fusobacterium* were significantly increased in patients with spondyloarthritis [208], indicating AS is potentially an oral microbiome-driven disease.

#### The oral microbiota and MS

MS is a chronic immune-mediated, inflammatory neurological disease that widely damages the brain and spinal cord, giving rise to demyelination and axonal and neuronal loss. The exact cause of MS has not been completely elucidated to date. Numerous studies have shown that the gut microbiome plays a critical role in the pathogenesis of MS [209]. However, the correlation between the oral microbiota and MS is still unknown. Compared with healthy controls, an increased abundance of *bacteroidetes* and *proteobacteria*, and a reduction in the levels of *firmicutes* and *actinobacteria* were found in the saliva specimens collected from monozygotic female twins with MS. At the genus level, *prevotella, rothia,* and *haemophilus* were depleted while *streptococcus* and *actinomyces* were over-represented in saliva specimens from MS patients [210]. Another study investigated that the salivary microbiome of MS is characterized increased abundance of the genera *Staphylococcus, Actinomyces, Fusobacterium, Bacteroides, Porphyromonas, Prevotella, Veillonella,* and *Propionibacterium* and uncultivable bacteria [211]. Lipid 654, a known product from *P. gingivalis* and functioned as a TLR2 ligand, potentially promotes inflammatory responses [212]. Therefore, we speculate that the detection of oral microbiota and its products may possibly have positive effect to identify the pathogenesis of MS and formulate preventive and therapeutic measures.

#### The oral microbiota and autoimmune liver diseases (AILDs)

AILDs are chronic inflammatory liver conditions that include AIH, PBC, and primary sclerosing cholangitis (PSC) [213]. AIH, a chronic immune-mediated and progressive condition, is characterized by elevated serum transaminases, hypergamma-globulinemia, and positive autoantibodies. The importance of gut microbiota in shaping and inducing the host immune response as well as promoting the development of AIH has been studied extensively [77, 214]. Abnormal gut microbiota composition in patients with AIH has been reported by Wei et al. The expression of obligate anaerobes was decreased, while *Veillonella* was increased in fecal samples from AIH patients. In addition, *Veillonella dispar* was positively associated with serum level of aspartate aminotransferase and the severity of hepatic inflammation. Moreover, abnormal lipopolysaccharide biosynthesis and amino acid metabolism were found in the fecal samples from AIH patients [215]. However, as oral microbiota plays a crucial role in shaping the composition and structure of gut microbiota, the role of oral microbiome in modulating the pathogenesis of AIH needs further investigations. In fact, the oral microbiome diversity was markedly increased in saliva samples from the AIH group compared with the healthy control group. The levels of *Streptococcus, Veillonella*, and *Leptotrichia* were significantly elevated in AIH patients [216]. In addition, AIH was characterized by an increase in *Veillonella* and a decrease *in Streptococcus* in the saliva samples. A positive correlation was found between the abundance of *Veillonella* and proinflammatory cytokine levels [217].

PBC is a chronic cholestatic liver disease that occurs predominant in women and is characterized by damage to small intrahepatic bile ducts. PBC may be driven by microbial imbalance, which leads to liver inflammation towards fibrosis and subsequent cirrhosis. A decreased abundance of *autochthonous* families and an increased abundance of *Enterobacteriaceae* and *Enterococcaceae* were observed in the saliva samples from cirrhotic patients [218]. Furthermore, Abe et al. showed that the levels of *Eubacterium* and *Veillonella* were increased in saliva samples from PBC patients, while that of *Fusobacterium* was decreased [219]. By using 16S rDNA sequencing, *Bacteroidetes, Campylobacter, Prevotella,* and *Veillonella* were found to be enriched in the saliva samples of PBC patients, while *Enterococcaceae, Granulicatella, Rothia,* and *Streptococcus* were depleted*.* In addition, saliva supernatants from PBC patients induced the production of inflammatory mediators, and the levels of inflammatory cytokines were significantly decreased following effective tooth brushing, indicating that a dysregulated oral microbiota may stimulate inflammatory responses to promote the development of PBC [220]. Of note, the oral microorganisms such as *Streptococcaceae* were found to be significantly increased in small intestinal lining in cirrhotic mice [221]. Due to the unique anatomical of hepato-enteric portal vein system, bacteria from oral cavity and gastrointestinal tract can translocate into liver tissues to trigger immune responses [222]. The immunosuppressive molecule, IL-35, was found to be lower in peripheral blood mononuclear cells of PBC patients than those of the healthy controls. Importantly, the levels of alkaline phosphatase and IFN-γ were negatively correlated with the level of plasma IL-35 in PBC patients [223]. Moreover, the expression of pro-inflammatory factors including IL-17, IL-6, IFN-γ, TNF-α and IL-10 was significantly higher in serum samples from PBC patients [224]. The bacterium might easily translocate into the portal blood and arrive at liver due to the breakdown of intestinal barrier [37].

PSC is a rare chronic cholestatic disease characterized by progressive inflammation and fibrosis of the intrahepatic and extrahepatic bile ducts. PSC might develop into cirrhosis, portal hypertension, and liver decompensation in the advanced stages [225]. Recently, Lapidot et al. reported that *Veillonella, Scardovia,* and *Streptococcus* were greatly enriched, while native bacteria were depleted, in the saliva samples from PSC patients by using 16S rRNA sequencing [226]. Furthermore, *Rothia* and *Haemophilus* were less abundant in saliva samples from PSC patients than in healthy controls [227]. By using 16S rRNA sequencing, significant differences in the bacterial community structure were found in the saliva samples from patients with PSC and healthy individuals [228]. Notably, it is estimated that approximately 70% of patients with PSC have IBD, which highlights the involvement of gut-liver axis [229]. It was found that the circulating bacterial translocation markers including lipopolysaccharide-binding protein and soluble CD14 were significantly elevated in the serum samples of patients with PSC [230]. Due to the assistance of impaired intestinal epithelial barrier, we speculated that oral microorganisms and their metabolites reached liver through hepato-enteric portal vein system and subsequently induce activation of immune cells.

#### The oral microbiota and immunoglobulin a nephropathy (IgAN)

IgAN is an immune-mediated and idiopathic glomerulonephritis characterized by mesangial hyperplasia with IgA deposition. The long-term prognosis for IgAN remains poor, with approximately 30–40% of IgAN patients developing end-stage renal failure within 10–20 years [231, 232]. To date, the immune triggers of IgAN are still not completely understood.

Accumulating evidence has demonstrated that the oral microbiome might be actively involved in the pathogenesis of IgAN. By using high-throughput sequencing technology, a dramatic increase in *Granulicatella* abundance and a marked reduction in *Prevotella* and *Veillonella* abundance were found in the saliva samples of IgAN patients [233]. Piccolo et al. demonstrated that the ratio of *Firmicutes to Proteobacteria* was significantly reduced, while the *Pasteurellacea*e family was found to be enriched, in saliva samples of IgAN patients [234]. Compared with healthy controls, the abundance of *Neisseria* was significantly higher in saliva samples from the IgAN group [235]. In addition, IgAN patients and healthy controls could be differentiated by the abundance *Capnocytophaga, Rothia*, and *Haemophilus* in saliva samples. In particular, the abundance of *Capnocytophaga* was found to be positively correlated with the level of proteinuria [236]. The changes in subgingival microbial structure seem to affect the incidence of IgAN. *Proteobacteria* and *Actinobacteria* at the phylum level, *Betaproteobacteria, Bacilli, Actinobacteria, Flavobacteriia,* and *Gammaproteobacteria* at the class level*,* and *Bergeyella, Capnocytophaga, Actinomyces, Corynebacterium, Comamonas, Lautropia,* and *Streptococcus* at the genus level were significantly abundant in the subgingival microbiome from CP patients with IgAN compared to those without IgAN. In addition, the bacterial composition in subgingival plaque differed greatly between CP patients with and without IgAN [237].

In particular, Cnm- ( +) *S. mutans* might be involved in the incidence and progression of IgAN [238]. First, Cnm- ( +) *S. mutans* was significantly abundant in saliva samples of IgAN patients. In addition, a higher Cnm protein-positive rate in tonsil specimens was observed in patients with IgAN [239]. Recently, Naka et al. reported higher positive staining rates of IgA and C3 in rats treated with Cnm- ( +) *S. mutans* [240]. More importantly, infection with *S. mutans* might promote the deposition of IgA1 in mesangial areas in glomeruli by abnormal glycosylation of serine or threonine amino acids of IgA1 [241]. The detailed alterations of oral microbial profile in various autoimmune diseases are summarized in Table 3.

**Table 3:** Alterations of oral microbial profile in autoimmune diseases

Autoimmune disease	Sample type	Screening methodology	Group	Alterations in oral microbial profile	References
IBD: UC and CD	Saliva	16S rRNA amplicon sequencing	22 IBD *vs*. 8 HCs	Increased: *Saccharibacteria* (TM7)*, Absconditabacteria* (SR1)*, Leptotrichia, Prevotella, Bulleidia,* and* Atopobium*	[133]
	Saliva	16S rRNA sequencing	54 UC, 13 CD *vs.* 25 HCs	UC: Increased of *Streptococcus and Enterobacteriaceae* species CD: Decreased of *Lachnospiraceae* and* Prevotella*	[134]
	Saliva	16S rRNA sequencing	29 CD *vs.* 31 HCs	Increased: *g. Prevotella, f. Prevotellaceae,* and* p. Bacteroidetes*	[137]
RA	Subgingival plaque	Shotgun metagenomics sequencing	48 positives for anti-CCP RA *vs.* 32 HCs	Increased: *P. gingivalis*	[154]
	Subgingival plaques	16S rRNA sequencing	54 RA *vs.* 44 HCs and 45 PD	Increased: *Porphyromonas, Prevotella,* and* Veilonella* Decreased: *Streptococcus, Gemella, Planobacterium*	[160]
	Subgingival plaque	16 S rRNA sequencing	8 RA and 10 non-RA	Increased: *S. parasanguinis *and* Actinomyces meyeri*	[162]
SLE	Saliva	16S rRNA sequencing	35 SLE *vs.* 35 HCs	Increased: *Veillonella, Streptococcus,* and* Prevotella*	[168]
	Mucosa swabs	16S rRNA sequencing	25 SLE *vs.* 19 HCs	Decreased: *Sphingomonadaceae, Halomonadaceae, and Xanthomonadaceae*	[12]
T1D	Oral swabs		53 T1D *vs* 50 HCs	Increased: *Streptococcus* spp*., Actinomyces* spp*. and Rothia* spp	[180]
	Saliva	16S rRNA sequencing	37 T1D *vs* 29 HCs	Increased: *Streptococcus*	[181]
	Subgingival	Bacterium-specific PCR	24 T1D *vs* 27 HCs	Increased: *Capnocytophaga sputigena and C. ochracea*	[183]
SS: PSS and SSS	Saliva	16S rRNA sequencing	9 pSS *vs.* 5 HCs	Increased: *Bacteroidetes and Actinobacteria;* Decreased: *Proteobacteria*	[197]
	Saliva	16S rRNA sequencing	37 pSS *vs.* 35 HCs	Increased: *Bifidobacterium, Dialister, and Lactobacillus* Decreased: *Leptotrichia*	[199]
	Supragingival plaque	CKB	37 SS *vs.* 35 HCs	Increased: *V. parvula, Fusobacterium nucleatum ss vincenti,* and* Propionibacterium acnes*	[200]
AS	Saliva	16S rRNA metagenomic sequencing	37 AS *vs.* 41 HCs	Increased: *Veillonellaceae, Brucella spp,* and* C. concisus* Decreased: *Streptococcus*	[207]
MS	Saliva	16S rRNA DGGE and Next generation sequencing	30 MS *vs.* 30 HCs	Increased: *Genera Staphylococcus, Actinomyces, Fusobacterium, Bacteroides, Porphyromonas, Prevotella, Veillonella, Propionibacterium* and* uncultivable bacteria*	[211]
AILDs: AIH, PBC, and PSC	Saliva	16S rRNA sequencing	68 AIH *vs.* 136 HCs	Increased: *Streptococcus, Veillonella,* and* Leptotrichia*	[216]
	Saliva	16S rRNA sequencing	56 AILD *vs.* 15 HCs	Increased: *Eubacterium and Veillonella* Decreased: *Fusobacterium*	[219]
	Saliva	16S metagenomic sequencing	39 PBS *vs.* 37 HCs	Increased: *Bacteroidetes, Campylobacter, Prevotella,* and* Veillonella* Decreased: *Enterococcaceae, Granulicatella, Rothia,* and* Streptococcu*	[220]
	Saliva	16S rRNA sequencing	35 PSC *vs.* 30 HCs	Increased: *Veillonella, Scardovia,* and* Streptococcus*	[226]
	Saliva	16S rRNA sequencing	24 PSC *vs.* 24 HCs	Decreased: *Rothia* and* Haemophilus*	[227]
IgAN	Saliva	16S rRNA sequencing	28 lgAN *vs.* 25 HCs	Increased: *Granulicatella;* Decreased: *Prevotella* and *Veillonella*	[233]
	Saliva	16S rRNA pyrosequencing	28 lgAN *vs.* 14 HCs	Increased: *Pasteurellacea*e Decreased: Ratio of *Firmicutes to Proteobacteria*	[234]
	Saliva	16S rRNA amplicon sequencing	43 lgAN *vs.* 65 HCs	Increased: *Neisseria*	[235]
	Saliva	16S rRNA sequencing	31 lgAN *vs.* 30 HCs	Decreased: *Capnocytophaga, Rothia*, and *Haemophilus*	[236]

*CD* crohn's disease, *PSS* primary sjogren syndrome, *SSS* secondary sjogren syndrome, *AS* ankylosing spondylitis, *MS* multiple sclerosis, *AILDs* autoimmune liver diseases, *AIH* autoimmune hepatitis; *PSC* primary sclerosing cholangitis, *IgAN* immunoglobulin a nephropathy. *HCs* healthy controls; Cm group, patients with both Crohn's disease and periodontitis, *Pm group* Patients with periodontitis; Hm group, Healthy individuals; *CKB* Checkerboard DNA-DNA hybridization, *PD* periodontitis. anti-CCP, Anti-cyclic citrullinated peptide, *DGGE* denaturing gradient gel electrophoresis. *T1D* type 1 diabetes.

#### Balancing the oral microbiota for treating autoimmune diseases

**Fig. 5:**
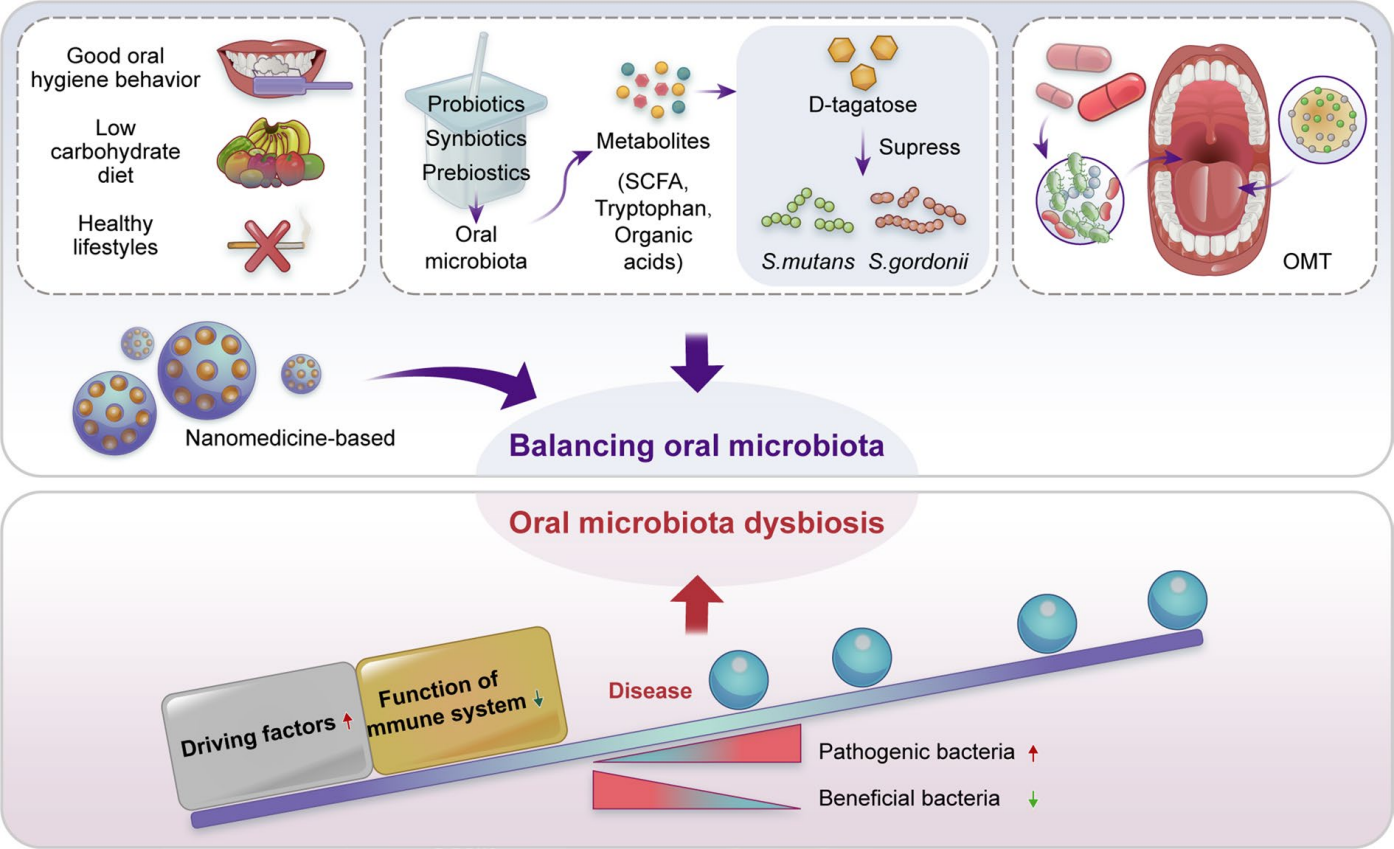
Treatment strategy on balancing oral microbiota for autoimmune diseases. Oral microbiota dysbiosis could lead to development of autoimmune diseases, therefore good oral hygiene behavior, low carbohydrate diet, healthy lifestyles, metabolites (prebiotics, probiotics, synbiotics), OMT, and Nanomedicine-based treatment are viable approaches to reverse dysbiosis and restore eubiosis. Increasing driving factors, such as good oral hygiene behavior, low carbohydrate diet, healthy lifestyles could reduce percentage of oral microbial dysbiosis. Prebiotics, probiotics, synbiotics could inhibit oral pathogenic microorganisms and prevent infections caused by oral pathogens. On the other hand, OMT introduces a new and healthy bacterial community to the recipient with the intent of reversing the established dysbiosis. Moreover, the combined effects of nanomedicine-based therapeutics and OMT could potentially and precisely use to oral microbiota delivery. OMT, oral microbiota transplantation; SCFA, Short-chain fatty acids

Due to the indispensable role of oral microbiota dysbiosis in the initiation and progression of autoimmune diseases, targeting the oral microbiota might be an effective strategy for preventing and treating autoimmune diseases (Fig. 5). Maintenance of the dynamic homeostasis between the oral microbiota and the host immune system contributes to maintaining body health. Avoiding the driving factors for oral microbiota dysbiosis and boosting the host’s immune system are two potential practical approaches for balancing the oral microbiota. Good oral hygiene behaviors, a low carbohydrate diet and healthy lifestyles reduce the oral microbial/bacterial load [242]. In addition, the usage of prebiotics, probiotics, or synbiotics not only reverses oral microbiota dysbiosis but also enhances the resilience of oral microflora [243, 244]. For instance, the prebiotic D-tagatose suppressed the growth of the oral pathogens *S. mutans* and *Streptococcus. gordonii* [245]. Interestingly, it has been demonstrated that prebiotics affect gut microbiota composition and enhance the production of beneficial microbial metabolites (short-chain fatty acids, tryptophan and organic acids) for pathogenic bacterial suppression and gut barrier improvement [246]. Targeting microbiota-derived metabolites might also be an effective strategy for treating oral microbiota-mediated autoimmune diseases.

Fecal microbiota transplantation (FMT) is an emerging strategy for the treatment of refractory diseases in recent years. For example, this approach has demonstrated promising effects in maintaining remission status in CD and inducing remission in UC [247, 248]. In addition, the gut microbiota could be modulated for the majority of participants by using oral FMT capsules from healthy lean donors [249]. We propose that oral microbiota transplantation (OMT) might be a potential effective strategy for treating oral microbiota-mediated autoimmune diseases, as OMT has shown great promise for curing oral microbiota-mediated diseases. For instance, the microbiota composition in dogs with naturally occurring periodontitis could be regulated by OMT [250]. In addition, OMT alleviated radiotherapy-induced oral mucositis by reshaping the oral bacteria taxonomic composition in mice [251]. Oral bacteria can be effectively encapsulated as a treatment for autoimmune diseases.

Enhancing the synergistic effects of balancing the oral microbiota might be important for achieving better therapeutic outcomes. TNF-α siRNA nanomedicine-targeted macrophages significantly attenuated TNF-α levels and improved IBD in an animal model [252, 253]. Further studies are warranted to determine the combined effects of nanomedicine-based therapeutics and OMT.

## Conclusions

It is now well recognized that oral microbiota dysbiosis plays a crucial role in modulating the initiation and development of many autoimmune diseases, and targeting oral microbes might be a promising strategy for ultimately achieving the goal of curing autoimmune diseases. Unfortunately, few clinical trials have evaluated the beneficial effects of microbiota-based therapies on autoimmune diseases. In addition, effective strategies for balancing the oral microbiota to treat autoimmune diseases are urgently needed. Moreover, the underlying molecular mechanisms by which host-microbe interactions in the oral cavity remotely trigger and exacerbate autoimmune diseases remain unclear. Systematic combination of metabolomics, metagenomics, metatranscriptomics, and proteomics might improve our understanding of the complex interactions between the oral microbial ecosystem and immune system, which might provide novel oral microbiota targets for treating autoimmune diseases.

## Data Availability

Not applicable.

## Abbreviations

PADs: Peptidylarginine deiminases
Aa: Aggregatibacter actinomycetemcomitans
LtxA: Toxin leukotoxin-A
SLE: Systemic lupus erythematosu
IBD: Inflammatory bowel disease
TLRs: Toll-like receptors
PRRs: Pattern recognition receptors
APCs: Antigen-presenting cells
DSS: Dextran sodium sulfate
MS: Multiple sclerosis
Amp: Ampicillin
Tyl: Tylosin
SS: Sjögren’s syndrome
PDC-E: Pyruvate dehydrogenase complex E2
PBC: Primary biliary cirrhosis
ACPAs: Anti-citrullinated protein antibodies
UC: Ulcerative colitis
CD: Crohn's disease
RA: Rheumatoid arthritis
SCWs: Streptococcal cell walls
vWFA: Von willebrand factor type a domain protein
AS: Ankylosing spondylitis
AILDs: Autoimmune liver diseases
AIH: Autoimmune hepatitis
PSC: Primary sclerosing cholangitis
IgAN: Immunoglobulin A nephropathy
FMT: Fecal microbiota transplantation
OMT: Oral microbiota transplantation
T1D: Type I diabetes
BF: Bacteroides fragilis
PAO: Periodontitis-associated microbiota-administered
HAO: Health-associated oral microbiota-administered
MHC: Major histocompatibility complex
HbA1c: Glycosylated hemoglobin
TJP: Tight junction protein
